# Multidisciplinary Approach in Managing Orbital Tumors in Ibadan, Nigeria

**DOI:** 10.4137/cmo.s757

**Published:** 2008-05-08

**Authors:** T.S. Oluleye, A.O. Fasola, O. Komolafe

**Affiliations:** 1 Lecturer and Consultant Ophthalmologist, Department of Ophthalmology, University College Hospital, PMB 5116, Ibadan, Nigeria; 2 Senior Lecturer and Consultant Oral and Maxillofacial Surgeon. Department of Oral and Maxillofacial Surgery, University College Hospital, PMB 5116, Ibadan, Nigeria; 3 Senior Registrar, Ophthalmology Department, University College Hospital, Ibadan

Orbitotomy is not a commonly performed surgical procedure in Nigeria due to paucity of trained orbitoplastic surgeons. Two cases of orbital tumors managed with lateral orbitotomy in conjunction with a maxillofacial surgeon are presented to stress the importance of multidisciplinary approach in managing these cases in the developing countries.

## Case 1

Patient A. O., an 8-year-old school pupil presented to the Eye Clinic of the University College Hospital, Ibadan with a 3 year history of gradual painless protrusion of the left eye. She lost vision in the eye 4 months before presentation.

**Figure 1:**
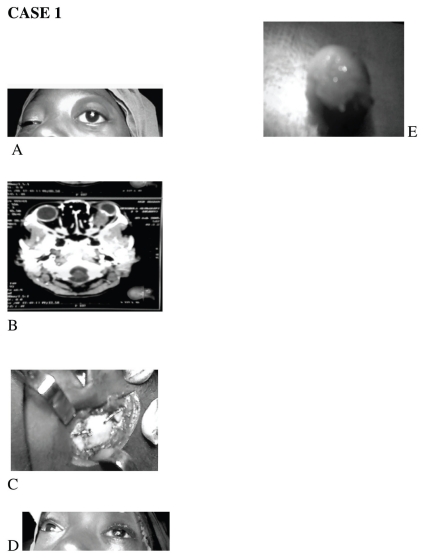
(**A**) Proptosis of the left eye (**B**) CT Scan showing left optic nerve enlargement (**C**) Intraoperatively, bone window secured with 0.40 mm stainless steel wires; (**D**) Immediate postoperatively, regression of proptosis of left eye. (**E**) Excised mass.

The general examination was normal. No features of neurofibromatosis were found. The visual acuity were 6/5 and NLP in the right and left eyes respectively. There was a non-axial proptosis of about 18 mm in the left eye. The deviation was down and out. No palpable mass was felt, and the orbital rim was smooth. The proptosis was not tender, non-retropulsive, non-pulsatile and no bruit was heard over the eyeball. The extra ocular muscle movements were full in all directions of gaze (Fig. 1A). An afferent pupillary defect was present in the left eye. Dilated fundoscopy showed left optic atrophy. The right eye was normal. A cranial CT scan showed a fusiform enlargement of the left optic nerve compressing the eyeball. No intracranial extension was seen. (Fig. 1B) An assessment of left optic nerve glioma was made. Differential diagnosis of unilateral proptosis in the young includes orbital cellulitis, orbital tumors such as Burkitt’s lymphoma, myeloid leukemia and secondaries. Further evaluation and investigations supported an optic glioma.

## Case 2

Patient A.B, 47 year old woman with 10 year history of gradual painless proptosis of the right eye. Examination showed right visual acuity of 6/12, non axial proptosis, superotemporal firm, non tender orbital mass, mild ophthalmoplegia, and choroidal folds on fundoscopy. The right optic disc was normal. Regional lymph nodes were not palpably enlarged. The left eye was normal at presentation. Cranial computerized tomography scan showed a circumscribed superolateral orbital mass, with no intracranial extension. An assessment of a lacrimal gland tumour was made.

**Figure 2:**
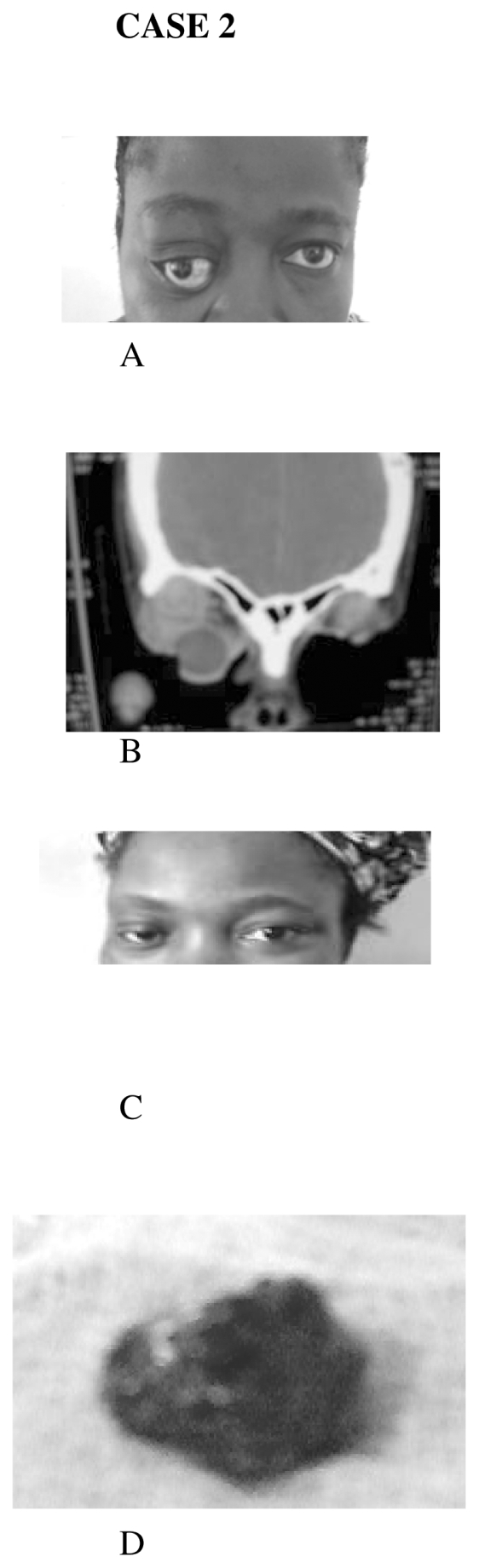
(**A**) Proptosis of the right eye (**B**) Coronal CT showing a superotemporal orbital mass (**C**) The same patient 3 months postoperatively (**D**) Excised mass.

The two patients had lateral orbitotomy in conjunction with the maxillofacial surgeons. At surgery, a C shaped skin incision was made just lateral to the lateral orbital rim, Hemostasis was secured. Subcutaneous tissue and temporalis muscle were dissected to gain access to the lateral orbital rim. Periosteal elevator was used to expose the fronto zygomatic suture, and with the aid of a drill, two bore holes were drilled on either sides of the suture to facilitate the removal of a bone window by a rotating electric saw (Fig. 1D). The periorbital was then incised to expose the orbital contents. The lateral rectus and orbital fat were retracted to expose the retroocular space. In the first case, a firm fusiform retroocular tumor, measuring 30 by 20 by 20 mm was excised and sent for histopathological examination (Fig. 1E). In the second case, a firm encapsulated mass was removed completely from the lacrimal fossa measuring 35 by 33 by 32 mm. It was also sent for histopathologic examination (Fig. 2D). The periorbital was closed and the bone fragment secured with surgical wire. The periosteum, temporalis muscle, subcutaneous tissue and skin were closed in layers. The proptosis regressed promptly (Figs. 1C and 2C). Hemostasis was secured by firm bandaging. Intravenous antibiotics and oral steroid were administered for infection prophylaxis and postoperative edema respectively.

All the instruments used were provided by the maxillofacial surgery department.

The sections of specimen from the first patient were in keeping with pilocytic astrocytoma grade I. In the second patient, the sections showed a malignant epithelial neoplasm consistent with adenocystic carcinoma of the lacrimal gland. Differential diagnosis of proptosis in an adult include other causes of lacrimal swelling such as dacryoadenitis, benign mixed lacrimal tumors, secondaries from breast in women and prostate in men. Investigations and examination did confirm a malignant neoplasm.

Patients had postoperative external beam radiotherapy.

## Discussion

Lateral orbitotomy has evolved over the years. It was first used in 1889^1^ and had since modified by various workers.^2^,^3^ It is less invasive than the transcranial approach and can be adapted to the developing country environment where facilities for invasive procedures are lacking.

A preoperative computerized tomography scan [CT scan] is advised in all cases to delineate the tumor and rule out intracranial extension. Other criteria used in determining whether the lateral approach is indicated include the location of the tumor, the size, and its pathology.^4^ In the two cases presented, CT scan helped in determining the extent of the tumors and excluding intracranial extension.

The maxillofacial surgeons created the access to the orbital contents by creating a bone window at the lateral wall of the orbit through an s-shaped incision lateral to the brow. The bone that was removed was subsequently replaced and secured to the adjacent bone with 0.40 mm soft stainless steel wires. The instruments used at this stage of the surgery are part of basic maxillofacial surgical set which are readily available in maxillofacial unit of hospitals in the developing countries. Other approaches to the orbit include Trans conjuctiva and intracranial approach. The Trans-conjuctiva route produces little room to work while the intracranial route produces more complications despite producing more exposure.

Reported complications of lateral orbitotomy include orbital hemorrhage, edema, optic nerve compression, orbital infection and lateral gaze palsy.^5^ The second patient developed deep vein thrombosis of the left lower limb 3 days post operatively. She was successfully managed with anticoagulation. It is recommended that patients should have early ambulation especially those at risk of developing DVT. Adequate cauterization, firm padding, post operative steroids and antibiotics helped in minimizing other complications.

Our center has no oculoplastic surgeon, hence the need for the general ophthalmologist to collaborate with other units in the management of such cases.
